# Crystal structure of 2,6-bis­[(1*H*-pyrazol-1-yl)meth­yl]pyridine

**DOI:** 10.1107/S1600536814017474

**Published:** 2014-08-06

**Authors:** Kyung-sun Son, Jeong Oh Woo, Daeyoung Kim, Sung Kwon Kang

**Affiliations:** a Department of Chemistry, Chungnam National University, Daejeon 305-764, Republic of Korea

**Keywords:** crystal structure, pyridine, purazole, tridentate ligand, catalysis

## Abstract

In the title compound, C_13_H_13_N_5_, the planes of the pyrazolyl groups are nearly perpendicular to that of the central pyridine ring, making dihedral angles of 87.77 (8) and 85.73 (7)°. In the crystal, weak C—H⋯N hydrogen bonds link the mol­ecules into layers extending parallel to (10-1).

## Related literature   

For the synthesis of the title compound, see: Reger *et al.* (2005 ▶). For metal complexes with similar ligands, see: Sharma *et al.* (2011 ▶); Ojwach *et al.* (2007 ▶); Manikandan *et al.* (2000 ▶, 2001 ▶); Halcrow & Kilner (2002 ▶). For potential applications of the ligand in catalysis, see: Karam *et al.* (2005 ▶).
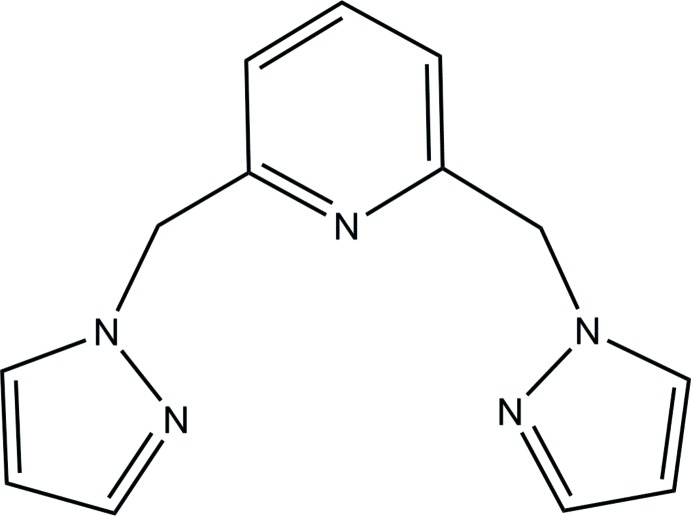


## Experimental   

### Crystal data  

C_13_H_13_N_5_
*M* *_r_* = 239.28
Monoclinic, 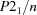

*a* = 7.481 (3) Å
*b* = 9.076 (4) Å
*c* = 19.021 (8) Å
β = 95.471 (5)°
*V* = 1285.7 (9) Å^3^
*Z* = 4
Mo *K*α radiation
μ = 0.08 mm^−1^
*T* = 296 K
0.26 × 0.2 × 0.15 mm

### Data collection  

Bruker SMART CCD area-detector diffractometer
25319 measured reflections
3136 independent reflections
2260 reflections with *I* > 2σ(*I*)
*R*_int_ = 0.040

### Refinement  

*R*[*F* ^2^ > 2σ(*F* ^2^)] = 0.060
*wR*(*F* ^2^) = 0.149
*S* = 1.09
3136 reflections
163 parameters
H-atom parameters constrained
Δρ_max_ = 0.24 e Å^−3^
Δρ_min_ = −0.28 e Å^−3^

### 

Data collection: *SMART* (Bruker, 2002 ▶); cell refinement: *SAINT* (Bruker, 2002 ▶); data reduction: *SAINT*; program(s) used to solve structure: *SHELXS2013* (Sheldrick, 2008 ▶); program(s) used to refine structure: *SHELXL2013* (Sheldrick, 2008 ▶); molecular graphics: *ORTEP-3 for Windows* (Farrugia, 2012 ▶); software used to prepare material for publication: *WinGX* (Farrugia, 2012 ▶).

## Supplementary Material

Crystal structure: contains datablock(s) global, I. DOI: 10.1107/S1600536814017474/is5371sup1.cif

Structure factors: contains datablock(s) I. DOI: 10.1107/S1600536814017474/is5371Isup2.hkl

Supporting information file. DOI: 10.1107/S1600536814017474/is5371Isup3.cml

**Fig. 1.**
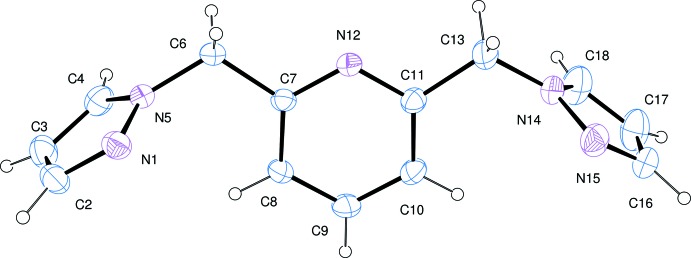
Molecular structure of the title compound, showing the atom-numbering scheme and 30% probability ellipsoids.

. DOI: 10.1107/S1600536814017474/is5371fig1.tif
Mol­ecular structure of the title compound, showing the atom-numbering scheme and 30% probability ellipsoids.

**Fig. 2.**
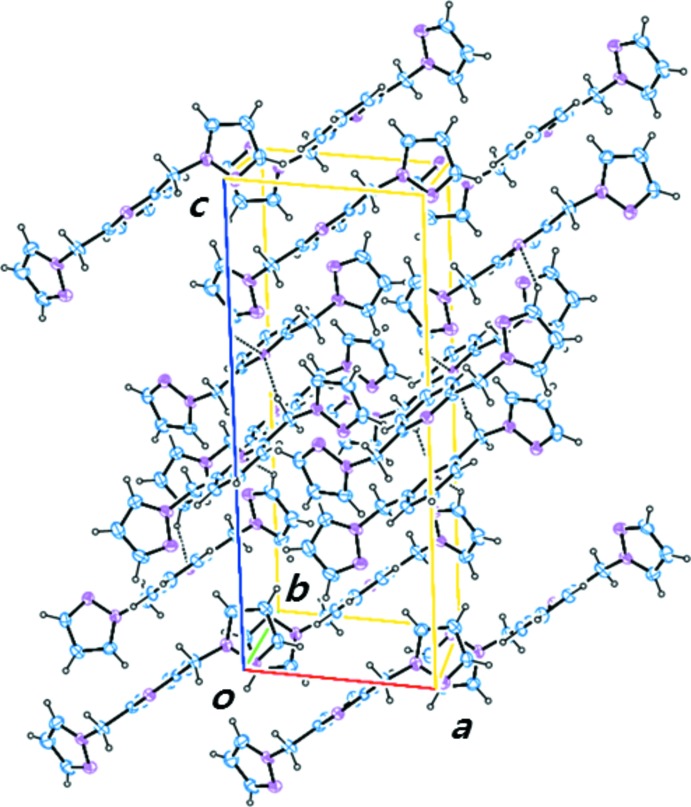
Part of the crystal structure of the title compound, showing molecules linked by intermolecular C—H···N hydrogen bonds (dashed lines).

. DOI: 10.1107/S1600536814017474/is5371fig2.tif
Part of the crystal structure of the title compound, showing mol­ecules linked by inter­molecular C—H⋯N hydrogen bonds (dashed lines).

CCDC reference: 1016859

Additional supporting information:  crystallographic information; 3D view; checkCIF report

## Figures and Tables

**Table 1:** Hydrogen-bond geometry (Å, °)

*D*—H⋯*A*	*D*—H	H⋯*A*	*D*⋯*A*	*D*—H⋯*A*
C4—H4⋯N15^i^	0.93	2.62	3.550 (3)	178
C6—H6*B*⋯N12^ii^	0.97	2.54	3.430 (2)	152

Symmetry codes: (i) 
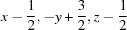
; (ii) 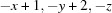.

## supplementary crystallographic information

### S1. Experimental

To a stirred solution of 2,6-pyridinedimethanol (0.28 g, 2 mmol) and NaOH (0.8 g, 20 mmol) in THF/water (7.5/7.5 ml) was added a solution of *p*-toluenesulfonyl chloride (0.761 g, 4 mmol) in THF (7.5 ml) at 0 °C. After 4 h of stirring, the mixture was poured into 20 ml of water and extracted with methylene chloride. The organic phase was washed with saturated aqueous NaCl solution and distilled water and dried over Na_2_SO_4_, and the solvent was removed *in vacuo* to afford 2,6-pyridine-dimethylene-ditosylate (0.788 g, 88%) as a white powder. In a separate flask under a nitrogen atmosphere, a solution of pyrazole (0.22 g, 3.2 mmol) in dry THF (5 ml) was added dropwise to a suspension of NaH (0.08 g, 3.2 mmol) in dry THF (5 ml) at 0 °C. After 15 min of stirring, a clear solution of NaPz was obtained. A solution of 2,6-pyridine-dimethylene-ditosylate (0.73 g, 1.6 mmol) in dry THF (7.5 ml) was added to this solution; the mixture was stirred overnight and filtered, and the solvent was removed. The crude product was purified by column chromatography on silica gel with ethyl acetate as eluent to afford 0.30 g (76%) of pure ligand as a white solid. Single crystals of the title compound were obtained by slow diffusion of hexane into a concentrated solution of the white solid in THF at room temperature within 1–2 days.

### S2. Refinement

All H atoms were positioned geometrically and refined using a riding model, with C—H = 0.93–0.97 Å, and with *U*_iso_(H) = 1.2*U*_eq_(C).

### Crystal data

**Table:**

C_13_H_13_N_5_	*F*(000) = 504
*M**_r_* = 239.28	*D*_x_ = 1.236 Mg m^−^^3^
Monoclinic, *P*2_1_/*n*	Mo *K*α radiation, λ = 0.71073 Å
Hall symbol: -P 2yn	Cell parameters from 5271 reflections
*a* = 7.481 (3) Å	θ = 2.2–25.5°
*b* = 9.076 (4) Å	µ = 0.08 mm^−^^1^
*c* = 19.021 (8) Å	*T* = 296 K
β = 95.471 (5)°	Block, colourless
*V* = 1285.7 (9) Å^3^	0.26 × 0.2 × 0.15 mm
*Z* = 4	

### Data collection

**Table:**

Bruker SMART CCD area-detector diffractometer	*R*_int_ = 0.040
Radiation source: fine-focus sealed tube	θ_max_ = 28.2°, θ_min_ = 2.2°
φ and ω scans	*h* = −9→9
25319 measured reflections	*k* = −12→12
3136 independent reflections	*l* = −25→25
2260 reflections with *I* > 2σ(*I*)	

### Refinement

**Table:**

Refinement on *F*^2^	0 restraints
Least-squares matrix: full	Hydrogen site location: inferred from neighbouring sites
*R*[*F*^2^ > 2σ(*F*^2^)] = 0.060	H-atom parameters constrained
*wR*(*F*^2^) = 0.149	*w* = 1/[σ^2^(*F*_o_^2^) + (0.0539*P*)^2^ + 0.4331*P*] where *P* = (*F*_o_^2^ + 2*F*_c_^2^)/3
*S* = 1.09	(Δ/σ)_max_ < 0.001
3136 reflections	Δρ_max_ = 0.24 e Å^−^^3^
163 parameters	Δρ_min_ = −0.28 e Å^−^^3^

### Special details

**Table:**

Geometry. All e.s.d.'s (except the e.s.d. in the dihedral angle between two l.s. planes) are estimated using the full covariance matrix. The cell e.s.d.'s are taken into account individually in the estimation of e.s.d.'s in distances, angles and torsion angles; correlations between e.s.d.'s in cell parameters are only used when they are defined by crystal symmetry. An approximate (isotropic) treatment of cell e.s.d.'s is used for estimating e.s.d.'s involving l.s. planes.

### Fractional atomic coordinates and isotropic or equivalent isotropic displacement parameters (Å^2^)

**Table:**

	*x*	*y*	*z*	*U*_iso_*/*U*_eq_	
N1	−0.0152 (2)	0.79070 (17)	0.01406 (8)	0.0502 (4)	
C2	−0.1286 (3)	0.7141 (2)	−0.02875 (11)	0.0576 (5)	
H2	−0.2403	0.6823	−0.0172	0.069*	
C3	−0.0626 (3)	0.6867 (2)	−0.09235 (11)	0.0637 (6)	
H3	−0.1184	0.6351	−0.1306	0.076*	
C4	0.1019 (3)	0.7512 (2)	−0.08738 (10)	0.0569 (5)	
H4	0.1821	0.7522	−0.1218	0.068*	
N5	0.12629 (18)	0.81324 (15)	−0.02326 (8)	0.0438 (4)	
C6	0.2836 (2)	0.88545 (19)	0.01027 (11)	0.0529 (5)	
H6A	0.2458	0.9661	0.0388	0.063*	
H6B	0.3512	0.9272	−0.026	0.063*	
C7	0.4057 (2)	0.78510 (17)	0.05647 (9)	0.0382 (4)	
C8	0.3819 (2)	0.63422 (19)	0.05958 (10)	0.0481 (4)	
H8	0.2873	0.5885	0.0327	0.058*	
C9	0.5017 (2)	0.5534 (2)	0.10339 (10)	0.0544 (5)	
H9	0.4889	0.4517	0.1065	0.065*	
C10	0.6403 (2)	0.62335 (19)	0.14246 (9)	0.0497 (4)	
H10	0.7221	0.5703	0.1724	0.06*	
C11	0.6554 (2)	0.77450 (18)	0.13620 (8)	0.0424 (4)	
N12	0.53963 (18)	0.85448 (15)	0.09409 (7)	0.0402 (3)	
C13	0.8035 (3)	0.8643 (2)	0.17501 (11)	0.0619 (5)	
H13A	0.8628	0.9211	0.1408	0.074*	
H13B	0.7503	0.9334	0.2059	0.074*	
N14	0.9367 (2)	0.77839 (17)	0.21661 (8)	0.0504 (4)	
N15	0.9129 (2)	0.7377 (2)	0.28293 (8)	0.0620 (5)	
C16	1.0538 (3)	0.6541 (3)	0.30142 (12)	0.0687 (6)	
H16	1.0754	0.61	0.3455	0.082*	
C17	1.1637 (3)	0.6396 (3)	0.24904 (14)	0.0860 (8)	
H17	1.2698	0.5859	0.25	0.103*	
C18	1.0849 (3)	0.7199 (3)	0.19552 (12)	0.0785 (7)	
H18	1.1269	0.7323	0.1514	0.094*	

### Atomic displacement parameters (Å^2^)

**Table:**

	*U*^11^	*U*^22^	*U*^33^	*U*^12^	*U*^13^	*U*^23^
N1	0.0469 (8)	0.0497 (9)	0.0545 (9)	−0.0040 (7)	0.0074 (7)	0.0014 (7)
C2	0.0445 (10)	0.0549 (11)	0.0728 (13)	−0.0086 (9)	0.0029 (9)	−0.0026 (10)
C3	0.0622 (13)	0.0643 (13)	0.0615 (13)	−0.0056 (10)	−0.0110 (10)	−0.0094 (10)
C4	0.0613 (12)	0.0639 (12)	0.0458 (10)	0.0064 (10)	0.0063 (9)	0.0065 (9)
N5	0.0406 (7)	0.0410 (8)	0.0486 (8)	−0.0031 (6)	−0.0016 (6)	0.0103 (6)
C6	0.0475 (10)	0.0387 (9)	0.0696 (12)	−0.0087 (8)	−0.0093 (9)	0.0157 (8)
C7	0.0357 (8)	0.0361 (8)	0.0435 (9)	−0.0018 (6)	0.0068 (7)	0.0050 (7)
C8	0.0437 (9)	0.0375 (9)	0.0616 (11)	−0.0065 (7)	−0.0034 (8)	0.0042 (8)
C9	0.0583 (11)	0.0314 (8)	0.0721 (13)	−0.0021 (8)	−0.0015 (9)	0.0070 (8)
C10	0.0550 (11)	0.0414 (9)	0.0512 (10)	0.0062 (8)	−0.0033 (8)	0.0083 (8)
C11	0.0476 (9)	0.0409 (9)	0.0385 (8)	0.0012 (7)	0.0025 (7)	0.0003 (7)
N12	0.0441 (8)	0.0340 (7)	0.0419 (7)	−0.0017 (6)	0.0015 (6)	0.0032 (6)
C13	0.0706 (13)	0.0468 (11)	0.0633 (12)	−0.0017 (9)	−0.0204 (10)	−0.0002 (9)
N14	0.0540 (9)	0.0551 (9)	0.0396 (8)	−0.0044 (7)	−0.0075 (7)	0.0015 (7)
N15	0.0639 (10)	0.0806 (12)	0.0411 (9)	−0.0067 (9)	0.0033 (8)	0.0023 (8)
C16	0.0755 (14)	0.0739 (14)	0.0521 (12)	−0.0096 (12)	−0.0173 (11)	0.0148 (11)
C17	0.0613 (14)	0.115 (2)	0.0787 (17)	0.0220 (14)	−0.0098 (13)	0.0017 (15)
C18	0.0617 (13)	0.121 (2)	0.0539 (13)	0.0083 (14)	0.0119 (11)	0.0059 (13)

### Geometric parameters (Å, º)

**Table:**

N1—C2	1.317 (2)	C9—H9	0.93
N1—N5	1.345 (2)	C10—C11	1.383 (2)
C2—C3	1.372 (3)	C10—H10	0.93
C2—H2	0.93	C11—N12	1.336 (2)
C3—C4	1.358 (3)	C11—C13	1.511 (2)
C3—H3	0.93	C13—N14	1.441 (2)
C4—N5	1.340 (2)	C13—H13A	0.97
C4—H4	0.93	C13—H13B	0.97
N5—C6	1.442 (2)	N14—C18	1.326 (3)
C6—C7	1.511 (2)	N14—N15	1.343 (2)
C6—H6A	0.97	N15—C16	1.319 (3)
C6—H6B	0.97	C16—C17	1.357 (3)
C7—N12	1.332 (2)	C16—H16	0.93
C7—C8	1.383 (2)	C17—C18	1.342 (3)
C8—C9	1.376 (2)	C17—H17	0.93
C8—H8	0.93	C18—H18	0.93
C9—C10	1.372 (2)		
			
C2—N1—N5	104.28 (15)	C8—C9—H9	120.1
N1—C2—C3	112.10 (18)	C9—C10—C11	118.44 (16)
N1—C2—H2	123.9	C9—C10—H10	120.8
C3—C2—H2	123.9	C11—C10—H10	120.8
C4—C3—C2	105.09 (18)	N12—C11—C10	122.49 (16)
C4—C3—H3	127.5	N12—C11—C13	113.78 (15)
C2—C3—H3	127.5	C10—C11—C13	123.73 (16)
N5—C4—C3	106.77 (18)	C7—N12—C11	118.43 (14)
N5—C4—H4	126.6	N14—C13—C11	114.41 (16)
C3—C4—H4	126.6	N14—C13—H13A	108.7
C4—N5—N1	111.76 (15)	C11—C13—H13A	108.7
C4—N5—C6	128.93 (17)	N14—C13—H13B	108.7
N1—N5—C6	119.07 (15)	C11—C13—H13B	108.7
N5—C6—C7	113.96 (14)	H13A—C13—H13B	107.6
N5—C6—H6A	108.8	C18—N14—N15	111.34 (17)
C7—C6—H6A	108.8	C18—N14—C13	127.23 (18)
N5—C6—H6B	108.8	N15—N14—C13	121.22 (17)
C7—C6—H6B	108.8	C16—N15—N14	103.57 (17)
H6A—C6—H6B	107.7	N15—C16—C17	112.7 (2)
N12—C7—C8	122.57 (15)	N15—C16—H16	123.7
N12—C7—C6	114.16 (14)	C17—C16—H16	123.7
C8—C7—C6	123.27 (15)	C18—C17—C16	104.5 (2)
C9—C8—C7	118.33 (16)	C18—C17—H17	127.7
C9—C8—H8	120.8	C16—C17—H17	127.7
C7—C8—H8	120.8	N14—C18—C17	107.9 (2)
C10—C9—C8	119.73 (16)	N14—C18—H18	126
C10—C9—H9	120.1	C17—C18—H18	126

### Hydrogen-bond geometry (Å, º)

**Table:**

*D*—H···*A*	*D*—H	H···*A*	*D*···*A*	*D*—H···*A*
C4—H4···N15^i^	0.93	2.62	3.550 (3)	178
C6—H6*B*···N12^ii^	0.97	2.54	3.430 (2)	152

Symmetry codes: (i) *x*−1/2, −*y*+3/2, *z*−1/2; (ii) −*x*+1, −*y*+2, −*z*.
