# Supplementary figures and images for: Crystal structure of 2,6-bis­[(1H-pyrazol-1-yl)meth­yl]pyridine
Source: Acta Crystallogr Sect E Struct Rep Online. 2014 Aug 6;70(Pt 9):o973. doi: 10.1107/S1600536814017474 (PMC4186076; doi:10.1107/S1600536814017474)

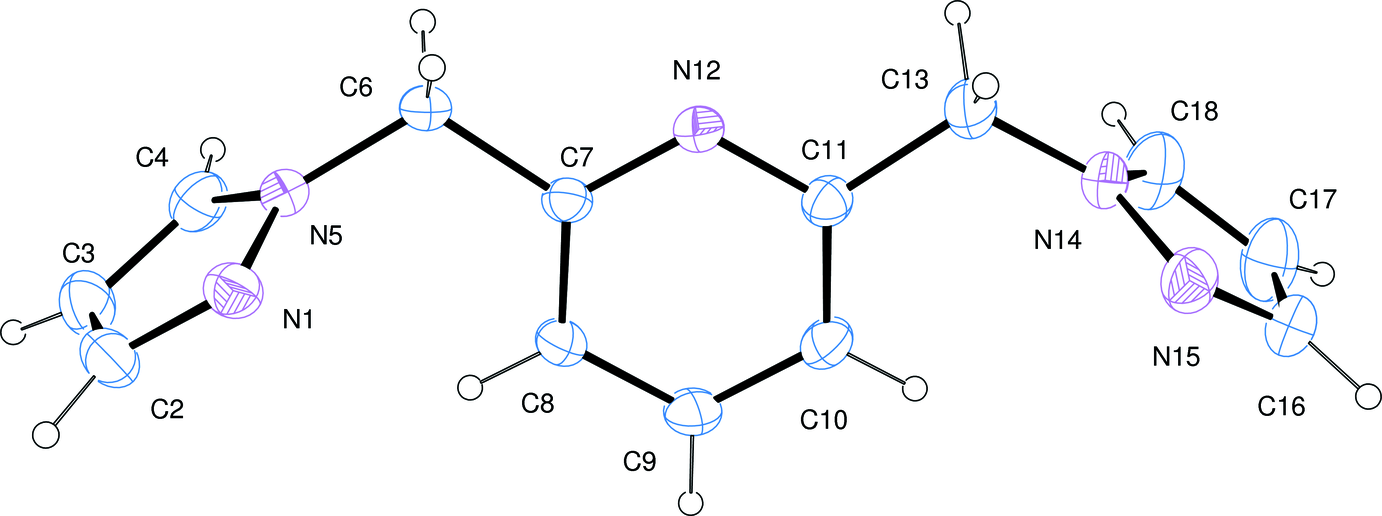

Supplement: Supplementary file 4 [file e-70-0o973-fig1.tif]

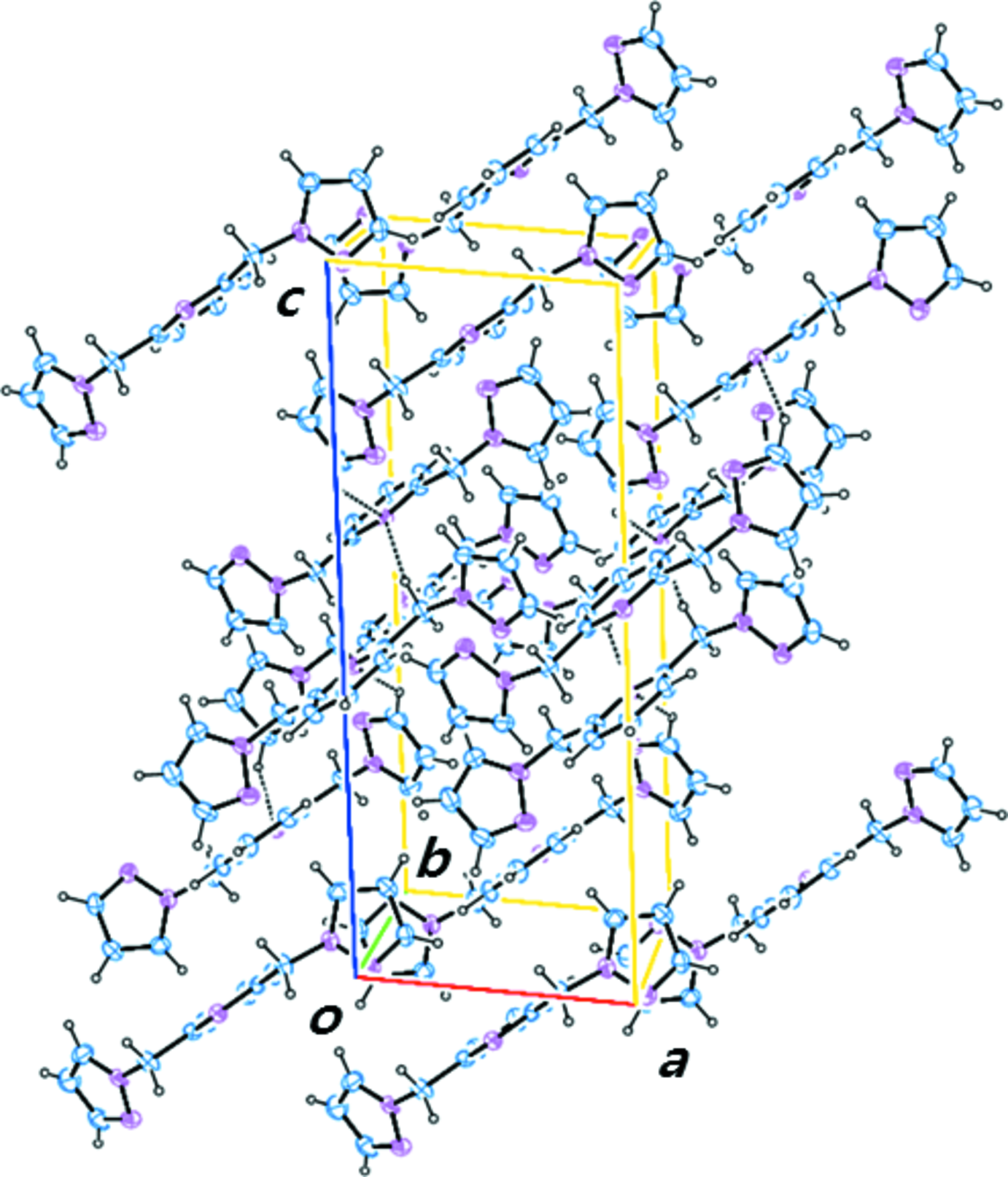

Supplement: Supplementary file 5 [file e-70-0o973-fig2.tif]
